# Imaging Mass Spectrometry Reveals Elevated Nigral Levels of Dynorphin Neuropeptides in L-DOPA-Induced Dyskinesia in Rat Model of Parkinson's Disease

**DOI:** 10.1371/journal.pone.0025653

**Published:** 2011-09-30

**Authors:** Anna Ljungdahl, Jörg Hanrieder, Maria Fälth, Jonas Bergquist, Malin Andersson

**Affiliations:** 1 Department of Pharmaceutical Biosciences, Drug Safety and Toxicology, Uppsala University, Uppsala, Sweden; 2 Department of Physical and Analytical Chemistry, Analytical Chemistry, Uppsala University, Uppsala, Sweden; 3 Unit Cancer Genome Research, Division of Molecular Genetics, German Cancer Research Center (DKFZ), Heidelberg, Germany; Emory University, United States

## Abstract

L-DOPA-induced dyskinesia is a troublesome complication of L-DOPA pharmacotherapy of Parkinson's disease and has been associated with disturbed brain opioid transmission. However, so far the results of clinical and preclinical studies on the effects of opioids agonists and antagonists have been contradictory at best. Prodynorphin mRNA levels correlate well with the severity of dyskinesia in animal models of Parkinson's disease; however the identities of the actual neuroactive opioid effectors in their target basal ganglia output structures have not yet been determined. For the first time MALDI-TOF imaging mass spectrometry (IMS) was used for unbiased assessment and topographical elucidation of prodynorphin-derived peptides in the substantia nigra of a unilateral rat model of Parkinson's disease and L-DOPA induced dyskinesia. Nigral levels of dynorphin B and alpha-neoendorphin strongly correlated with the severity of dyskinesia. Even if dynorphin peptide levels were elevated in both the medial and lateral part of the substantia nigra, MALDI IMS analysis revealed that the most prominent changes were localized to the lateral part of the substantia nigra. MALDI IMS is advantageous compared with traditional molecular methods, such as radioimmunoassay, in that neither the molecular identity analyzed, nor the specific localization needs to be predetermined. Indeed, MALDI IMS revealed that the bioconverted metabolite leu-enkephalin-arg also correlated positively with severity of dyskinesia. Multiplexing DynB and leu-enkephalin-arg ion images revealed small (0.25 by 0.5 mm) nigral subregions with complementing ion intensities, indicating localized peptide release followed by bioconversion. The nigral dynorphins associated with L-DOPA-induced dyskinesia were not those with high affinity to kappa opioid receptors, but consisted of shorter peptides, mainly dynorphin B and alpha-neoendorphin that are known to bind and activate mu and delta opioid receptors. This suggests that mu and/or delta subtype-selective opioid receptor antagonists may be clinically relevant for reducing L-DOPA-induced dyskinesia in Parkinson's disease.

## Introduction

The dopamine (DA) precursor L-DOPA is still the most effective symptomatic treatment in patients with Parkinson's disease (PD), but with time this treatment often causes troublesome complications, including “wearing off” fluctuations and peak-dose L-DOPA-induced dyskinesia (LID) [1]. Today many patients receive DA-agonists instead of L-DOPA as a first line of treatment of PD in order to reduce the likelihood and delay the development of LID. However, with time L-DOPA is still required for adequate symptomatic relief and thus patients suffer a greater risk of developing LID [2].

Evidence from both clinical studies and experimental models of Parkinson's disease link LID to disturbed opioid transmission in the basal ganglia [3], [4]. Several clinical and preclinical studies have aimed to reduce dyskinesia by using different opioid receptor antagonists and agonist, but results have been contradictory and difficult to interpret [4], [5], [6], [7], [8], [9], [10], [11], [12], [13]. In the rat model of LID in experimental PD, the severity of dyskinesia correlates strongly with striatal prodynorphin (PDyn or PPE-B) mRNA levels [14]. But so far no studies have assessed the possible association of LID with the different prodynorphin peptide products and their metabolites in the basal ganglia output structures that includes the substantia nigra (SN).

The exact nature of dynorphin peptides and their metabolites in the basal ganglia have mostly been studied by means of radioimmunoassay and immunohistochemistry, which rely on antibodies to the known molecular species of endogenous dynorphins and are hampered by possible cross-reactivity with closely related unknown peptide species [15], [16], [17], [18]. The prodynorphin propeptide can be processed into several different neuroactive peptides and several dynorphins have been detected in the rat substantia nigra by radioimmunoassay; including dynorphin A (Dyn A 1-8; Dyn A 1-17), dynorphin B (DynB), alpha- and beta-neoendorphin (aNeo, bNeo), and leu-enkephalin (Leu-Enk) [19], [20].

**Figure 1:**
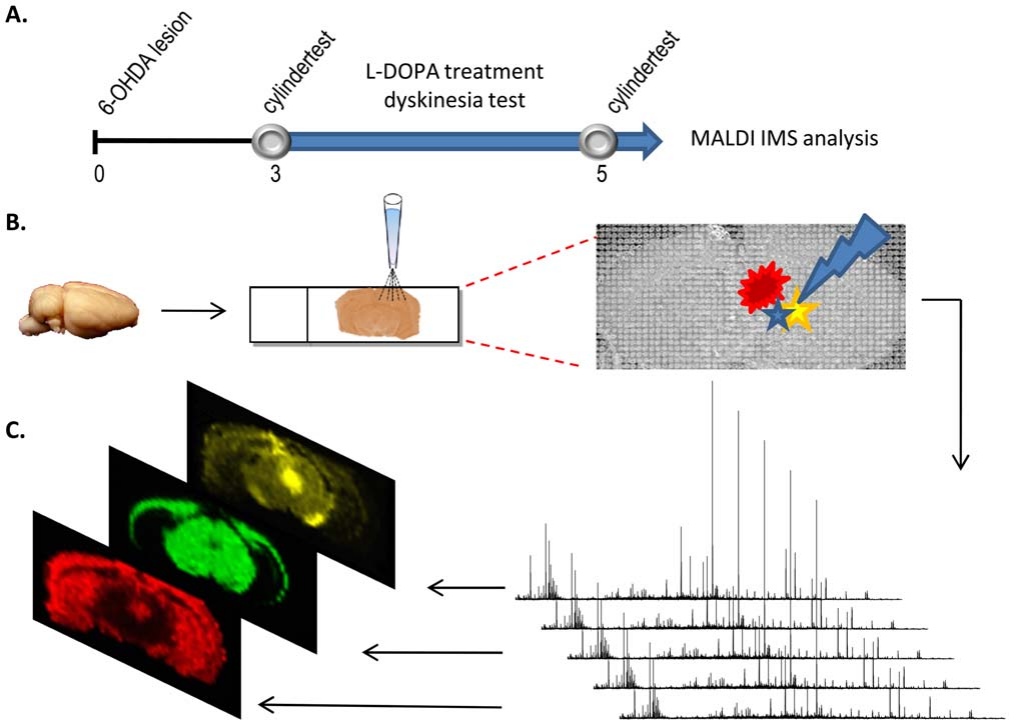
Experimental design. (A) The experimental set-up consist of unilateral injections of 6-OHDA into the right medial forebrain bundle anterior of the substantia nigra. A so-called cylinder test was used to assess forelimb asymmetry use and indicated the degree of DA-denervation. L-DOPA treatment and dyskinesia rating (blue arrow; 8 mg/(kg and day) commenced one day after the first cylinder test and ended one day after the second test. (B) The brains were dissected out 60 minutes after the last L-DOPA dose. Sections cut on a cryostat were thaw mounted onto MALDI compatible glass slides and 80 pL-sized drops of MALDI matrix placed in arrays across the sections. (C) Mass spectra were collected from every matrix spot and assembled into a MALDI imaging MS file that was used to visualize the distribution of different peptides, lipids and small proteins (displayed in yellow, green, and red).

In the current study, a unilateral rat model of PD was treated with a relative low dose of L-DOPA (8 mg/(kg and day) that is known to induce LID in some but not all animals. A mass spectrometry (MS) based imaging technique, MALDI-TOF imaging MS (MALDI IMS) was used for the detection of neuropeptides with high molecular weight accuracy and to determine their distribution in native tissue [21], [22] (experimental set up Fig. 1). For the first time it is possible to demonstrate alterations in endogenous prodynorphin-derived peptide levels in the substantia nigra of a model of LID in experimental PD.

## Methods

### Lesion surgery

Female Sprague-Dawley rats (Scanbur BK AB, Sweden), weighing 210 to 250 g at the time of surgery were housed four to a cage in a room with a 12-h light/dark cycle with food and water *ad libitum*. The experiments were approved by the Uppsala animal ethical committee (no 140/8) and followed the guidelines of Swedish legislation on animal experimentation (Animal Welfare Act SFS1998:56) and European Union legislation (Convention ETS123 and Directive 86/609/EEC). Animals were deeply anesthetized with isoflurane (Apoteksbolaget AB, Sweden). The toxin 6-OHDA-HCl (Sigma-Aldrich, Germany) was dissolved in 0.02% ascorbate saline to a concentration of 3.6 µg/µl and injected at the following coordinates in mm relative to bregma and the dural surface into the right ascending medial forebrain bundle [23]: (1) anterior -4.4; lateral 1.2; ventral 7.8; tooth bar at -2.3 (7.5 µg deposit); (2) anterior -4.0; lateral 0.8; ventral 8.0; tooth bar at +3.4 (6 µg deposit). Rimadyl (5 mg/kg, s.c., Apoteksbolaget AB) was used for post-operative analgesia.

### Behavioral tests

The degree of unilateral Parkinsonism was evaluated using the so-called cylinder test for forelimb-asymmetry use [24], [25]. At 3 weeks post lesion, the rats were placed in a glass-cylinder in a new environment and their forelimb touches to the glass wall (summing up to 20) were counted as they were rearing to explore the new environment. Only rats that used their contralateral forelimb less than 30% of the time were included in this study, corresponding to approximately 70% striatal DA depletion [25]. L-DOPA treatment and dyskinesia rating started the day after the limb-asymmetry test. The rats received daily L-DOPA (Sigma-Aldrich) (8 mg/kg) and benserazide (Sigma-Aldrich) (15 mg/kg) or physiological saline i.p. injections for 15 days and dyskinesia was rated by a blinded observer every other day according to the dyskinesia rating scale of Cenci et al. [14]. The rats were observed in individual cages every 20th minute (1-min monitoring periods) according to a scale of 0 to 4 for each four categories: 1) limb dyskinesia, 2) axial dystonia, 3) orolingual movements and 4) rotational/locomotor behavior. A score of “0” indicate the absence of abnormal movements. “1” indicates infrequent dyskinesia that occurred <50% of the time. “2” indicates occasional or frequent dyskinetic movement occurring >50% of the time. “3” specify an incident occurring constantly but can be interrupted and “4” refer to a constant, uninterruptible dyskinetic movement.

Forelimb asymmetry use was evaluated a second time, right before the last and 24 h after the previous L-DOPA administration.

#### Pdyn knockout mice

Pdyn^–/–^ mice were originally obtained by targeted deletion of the coding exons of the prodynorphin gene and bred into a 129SvEv-Tac background as described [26]. Animals were housed in groups of up to four per cage with food and water ad libitum in a room with a 12: 12 h light : dark cycle. DNA from each animal was extracted for subsequent genotyping to confirm the identity of the Pdyn locus as described [26] Brain tissues were isolated from 2 male and 1 female Pdyn knockout mice following overexposure to halothane and cervical dislocation, and frozen in liquid nitrogen.

### Tissue preparation

60 minutes after the last L-DOPA or saline injection the rats were deeply anesthetized with isoflurane and decapitated. The brains were rapidly removed (<30 seconds) and frozen on finely ground dry ice. Sections for immunohistochemistry were cut at 20 µm and thaw mounted on microscope slides (SuperFrost Plus; Menzel Glazer, Germany) whereas sections for MALDI-IMS analysis were cut at 12 µm and thaw mounted on to indium−tin oxide (ITO)-coated slides (Bruker Daltonics, Germany). The sections were dehydrated in a vacuum dessicator and stored at −80°C.

### Immunohistochemistry

Prior to immunohistochemistry the sections were immersed in phosphate buffered 4% paraformaldehyd for 2 h at RT, rinsed 3 times in tris-buffered saline and incubated in RTU blocking solution for 1 h. Immunohistochemistry (TH antiserum diluted 1∶1000, MAB318, Millipore, CA; Dyn B, 1∶5000, [18] was performed using a standard peroxidase-based method (RTU Vectastain kit, Vector laboratories, Burlingame, CA) and 3, 3′-diaminobenzidine (Vector laboratories) as chromogen, or using a fluorescent secondary antibody (Alexa 488, Invitrogen Co, CA).

Counts of TH-immunopositive cells at three rostrocaudal levels of SN were carried out by a blinded investigator.

For quantification of dynorphin immunoreactivity the optical density of the substantia nigra (medial and lateral) was analyzed with Image J (National Institutes of Health, Bethesda, MD).

### MALDI imaging mass spectrometry

In order to fixate tissue and remove salts that interfere with MALDI, the sections were washed once in 70% (10 seconds) and twice in 95% ethanol (2 times 10 seconds). The sections were dried face up in a vacuum desiccator. Matrix (50 mg/ml 2,5-di-hydroxybezoic acid (DHB) in 50% methanol, 15 mM ammonium acetate, and 0.3% trifluoroacetic acid (TFA), Sigma-Aldrich)) was applied using a chemical inkjet printer (CHIP-1000, Shimadzu Biotech, Japan) and droplets were placed in arrays over SN at a spatial resolution of 250 µm by 250 µm. The number of droplets and passes were optimized to 10 drops per pass for 10 passes. Mass spectra were acquired using an Ultraflex II (Bruker Daltonics, Germany), equipped with smart beam technology, operating in reflector positive mode. Each MALDI-adaptor was externally calibrated using a standard peptide mix (Peptide Calibration Standard II, Bruker Daltonics). All spectra were processed for baseline correction (Convex Hull) and exported as .dat-files using FlexAnalysis (Bruker Daltonics). Total ion current (TIC) normalization was performed on each individual spectrum [27]. Using Matlab (version R2009b) algorithm msalign and mspeaks available in Matlab bioinformatic toolpack all spectra were aligned and peaks with a singal-to-noise ratio >3 were detected. Binning was performed using a mass spectrometry peak binning software pbin [28] and the peak area AUC calculated for each peak using an in-house developed Matlab script. Data is expressed as average peak area for each area of interest. Animals displaying high degree of freeze damages to the sections or an uneven TIC, before normalization, were removed from data analysis, resulting in a total of 14 duplicate sets of sections per animal (n = 4 in LC group, n = 5 and 5 in LD and HD group).

### Neuropeptide identification

Opioid peptides were characterized in striatal extracts using a modified protocol based on strong cation exchange prefractionation as described previously [16], [29]. Briefly, frozen rat brain was mounted on a cryostat chuck at −20°C, the cortex and corpus callosum removed and the nucleus accumbens was separated from the caudate putamen by a thin cut from the most ventral point of the lateral ventricle to the most ventral point of the external capsule. Tissue sections (20 µm, n = 50 and 50, respectively) were thaw-mounted on separate superfrost glass slides (Thermo Scientific, Waltham, MA). Off tissue extraction was performed by repeated addition and collection of 300 microlitre 5% ACN, 0.1% TFA. The combined extracts were centrifuged at 5000 g for 10 min. The supernatant was collected, applied to small ion exchange columns (SP C25 Sephadex, GEHealthcare, Uppsala, Sweden), and eluted stepwise with buffers containing pyridine in 0.1% formic acid (0, 30, 70, and 100% pyridine). The fractions were dried down under reduced pressure using a speedvac system (ThermoSavant, Waltham, MA) and stored at −20°C. Prior to further analysis the fractions were reconstituted in 50 µL 0.1% formic acid.

Nanoscale liquid chromatography coupled to electrospray ionization Fourier transform ion cyclotron mass spectrometry (FTICR MS) was performed on an Agilent 1100 nanoflow system (Agilent Technologies, Santa Clara, CA, USA) hyphenated to a LCQ-FT 7.0 (Thermo Scientific). A volume of 5 µL from the reconstituted SCX fraction was injected automatically and loaded onto a C18 PicoFrit column (75 µm ID/15 µm tip ID, NewObjective, Woburn, MA) packed directly inside the electrospray needle tip using specially designed nanospray emitter tips. A water/formic acid (FA)/acetonitrile (ACN) solvent system was used where solvent A was 0.1% FA and solvent B was 100% ACN, 0.1% FA. Gradient elution was performed: 0% B for 10 min, 0% B to 50% B for 100 min, 50% B to 90% B for 5 min, 90%B for 5 min. Peptide elution was followed by ESI FTICR MS and tandem mass spectrometry (MS/MS) for peptide sequencing. A fullscan spectrum was acquired at high resolution (FWHM = 100000) using the FT analyzer. Data dependant acquisition was applied for MSMS precursor selection, where the 5 most intense mass peaks were subjected to subsequent isolation and collision-induced fragmentation in the ion trap. Annotated fragment spectra were subjected to a database containing sequences of known rat neuropeptides (SwePep peptides, 245 peptide entries, www.swepep.org,) and neuropeptide precursor proteins (Swepep precursor, 123 protein entries) using the Mascot search engine (v 2.2, matrixscience, London, UK). For peptide identification the search was made with the following specifications: no enzyme specificity; 10 ppm precursor tolerance and 1.2 Da fragment tolerance; no fixed modifications; variable modifications: C-term. amidation, deamidation, oxidation (M); precursor charges: 2+ and 3+; instrument: ESI trap. Peptide matches with a score above the confidence threshold (p<0.05) were considered to be a significant hit. The false positive identification rate (FPR) was estimated by searching the data against a decoy database, where the FPR threshold was set to <1%.

### Data analysis

Dyskinesia was evaluated using repeated measurement analysis of variance (ANOVA), between group differences at particular time points were tested by Bonferroni post hoc test.

For MALDI IMS data analysis, data was log2 transformed before further statistical analysis and a linear model was fitted using both treatment and analyses run as variables. For multiple testing correction of the p-values the [30] was used. An adjusted p-value <0.05 was considered as significant. Statistical analysis was done using the statistical environment R[31] and the package Limma [32]. Linear regression was used for its representative value but Pearson correlation was used to ascertain the nature of the relationship.

For all statistical comparisons including more than two groups one-factor ANOVA followed by Tukey *post hoc* test was used. The null hypothesis was rejected at p<0.05.

## Results

### The severity of dyskinesia increases over time

**Figure 2:**
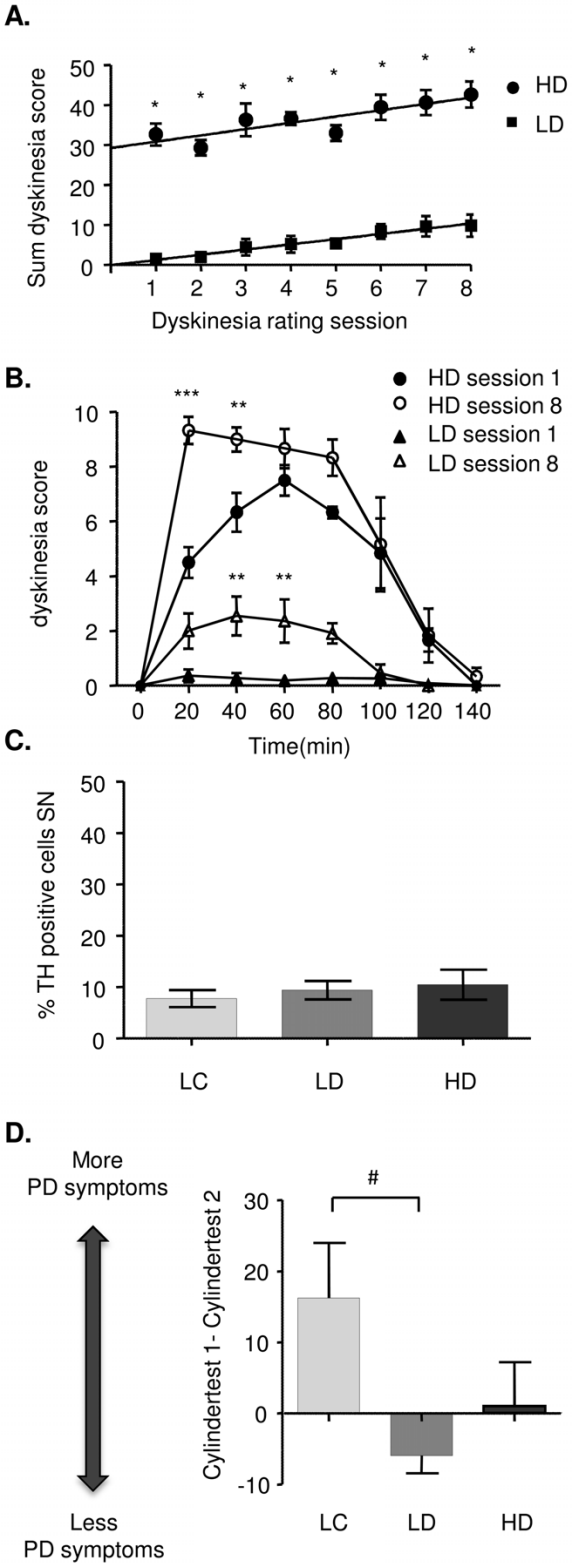
L-DOPA induced dyskinesia. (A) Two well-separated groups were observed, one high-dyskinetic (HD) and one low-dyskinetic (LD) group. The rate of dyskinesia development was similar in both groups, but the on-set was distinctly higher in the HD group. (B) The increase of dyskinesia score was mainly due to a shorter-onset in the HD group, and an increase in peak-dose severity in the LD group. (C) The difference in propensity to develop dyskinesia did not dependent on the number of remaining TH-immunopositive cells in the lesioned substantia nigra and was not significantly different between the two groups or lesion-only controls (LC group) (mean ± SEM, expressed as % of TH-immunoreactive neurons in the intact substantia nigra). (D) Low dyskinetic animals improved the left forelimb asymmetry use in the second cylinder test, whereas high-dyskinetic animals performed the same and lesion-only controls worsened. Data is expressed as the difference between the first and the second cylindertest (*p<0.01 HD vs. LD group, **p<0.01 session 8 vs. session 1 in HD and LD group, respectively, ***p<0.001 session 8 vs. session 1, HD group only, # p = 0.01 LC vs. LD).

Several rats displayed moderate to high LID on the first L-DOPA administration and two well separated groups could be observed; one high dyskinetic group (HD group, n = 6) and one low dyskinetic group (LD group, n = 11). The rate of dyskinesia development per day was similar in both groups (y = mx+b where m = 0.68±0.15 and 0.81±0.23 for LD and HD group respectively), but the offset was approximately 70 times higher in the HD group (b = 0.41 and 29.94 for LD and HD group respectively; Fig. 2A). Across the eight dyskinesia scoring sessions the severity of LID increased in both the LD and the HD group (p<0.0001 for time effect; p<0.0001 for group effect, no time x group interaction). Post hoc test revealed significant differences between the HD and LD group at all time points (p<0.001 HD vs. LD). Interestingly, even though LD animals displayed a higher peak-dose dyskinesia score at the end of treatment, no LD animal became severely dyskinetic during the treatment period (transit from LD to HD group). From the derived linear equation it may be possible to predict that it would take on average 42 days using 8 mg/(kg and day) L-DOPA for a low dyskinetic rat to become high dyskinetic.

Comparing the time course of dyskinesia of the first and last session revealed that the increase in dyskinesia severity had developed differently in the LD compared with the HD group (Fig. 2B, p<0.001 for time x treatment interaction). Post hoc analysis showed that the peak dose LID score had increased 8-fold in low dyskinetic rat at 40 and 60 minutes after injection (p<0.01 LD session 8 vs. LD session 1). By contrast, the increase in the HD group was mainly due to shortened onset latency (p<0.001 HD session 8 vs. HD session 1).

### Low dyskinetic rats show improvements of left forelimb use

The extent of the lesion was equal in all groups with less than 10% of the number of TH-immunopositive cells remaining in the lesioned compared with the intact control SN (Fig. 2C). DA-denervation by injection of the toxin 6-hydroxydopamine (6-OHDA) into the right medial forebrain bundle is associated with asymmetric use of the contralateral forelimb. This can be assessed by placing a rat in a transparent cylinder and registering the number of occasions that the rat uses the left or right paw to support its body as it rears to explore a new environment. In the hemi-parkinsonian rat model used in the present study the left paw is used to a lesser extent, which is expressed as a percent of the total number of left and right paw touches to the walls [24]. Neither the %left forelimb use before L-DOPA nor the number of TH-positive neurons correlated with dyskinesia severity, confirming other reports that the DA-denervation is a necessary but not sufficient factor for predicting the rats' susceptibility to develop dyskinesias (Putterman et al., 2007).

Forelimb asymmetry use was evaluated a second time before the last and 24 h after the previous L-DOPA administration. Interestingly, the %left forelimb use displayed a weak but significant negative correlation with the cumulative dyskinesia score (data not shown; p<0.05, Pearson's correlation coefficient R = 0.52). Comparing the first and last forelimb asymmetry test revealed that the low-dyskinetic group displayed less forelimb asymmetry at the second test, whereas the high-dyskinetic group showed no change and the lesion-only control (LC) group performed worse than during the first test (p<0.05, LD vs. LC; Fig. 2D).

### MALDI imaging mass spectrometry

**Figure 3:**
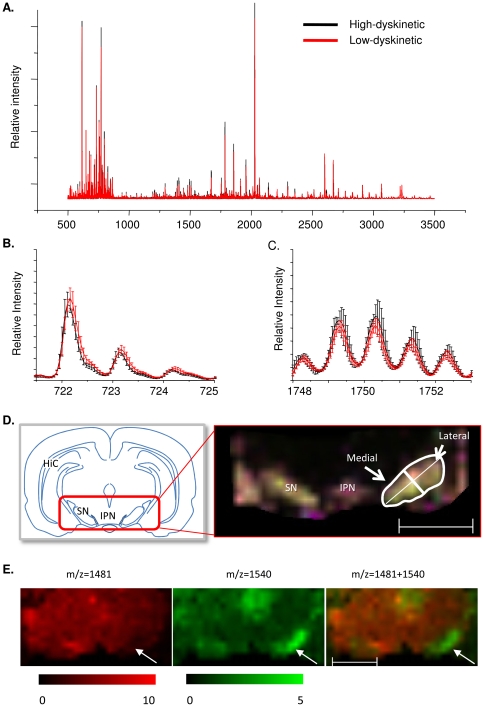
Imaging mass spectrometry. (A) MALDI IMS detects about 3000 peaks throughout the mass range scanned, between 500 to 3500 daltons, corresponding to around 1000 different monoisotopic molecular species. (B,C) Good MALDI IMS reproducibility is displayed as average mass spectra traces from the intact substantia nigra of low- and high-dyskinetic groups (mean ± SEM, n = 5 and 5 in LD and HD group, respectively). (D) The ventral midbrain was scanned with MALDI IMS and several different ions were multiplexed in order to define the area of the SN and to divide it into a medial and a lateral half, by bisecting a line from the ventromedial to the dorsolateral corner of SN. (E) MALDI IMS ion images of two examples of peptides that displayed a lesion-induced downregulation (red, m/z 1481) or upregulation (green, m/z 1540) in the whole SN (arrows). Abbreviation: HiC, hippocampus; IPN, interpeduncular nucleus; SN, substantia nigra. Scale bar  = 2 mm.

The MALDI IMS experiment was repeated twice on consecutive sections and over 1000 monoisotopic molecular species (corresponding to more than 3000 peaks) were detected in the mass range from 500–3500 Da (Fig. 3A). An overall coefficient of variance of 30% peak area intensities was observed for all peaks, but many peaks displayed very low variation (Fig. 3B). For analysis the SN was divided in 2 halves along a medial-lateral axis resembling two regions of interests (ROI, Fig. 3C). In each experiment changes in hundreds of peaks were observed in SN on the side ipsilateral to the 6-OHDA injection (lesion) relative to the contralateral (intact) side. For example both decreased (m/z 1481) and increased peak intensities (m/z 1540) were detected (Fig. 3D). Here, we focus on LID-associated changes of dynorphin-related peptides and only significant changes that occurred consistently in both experiments are reported. Many, but not all, endogenous dynorphin peptides have previously been identified by MS/MS analysis from murine brain tissue, including; Dyn A(1-8), aNeo and Leu-Enk-Arg [33], [34]. LC-MS/MS analysis of peptide extracts was performed in order to obtain peptide identities. Using high mass accuracy Fourier transform ion cyclotron mass spectrometry in conjunction with collision activated fragmentation several dynorphins, including Dyn B could be unequivocally identified according to its fragment mass data (Supplementary Fig. S1).

### MALDI IMS reveals that dynorphin B peak intensity correlates with dyskinesia severity

**Figure 4:**
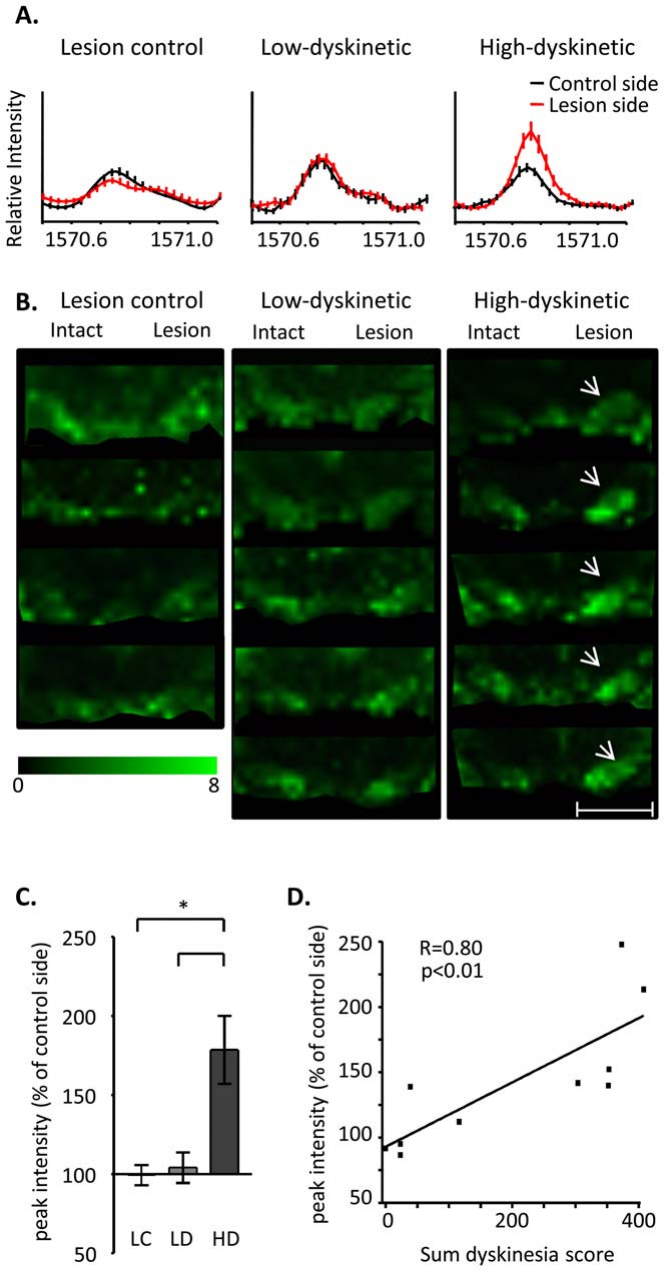
Dynorphin B peak intensities are elevated in the lateral SN of high dyskinetic group. (A) Average MS traces show a higher dynorphin B peak in HD group (average MS ± SEM, n = 5 and 5 in LD and HD group, respectively). (B) MALDI IMS of DynB ion images of animals from LC (n = 4), LD (n = 5) and HD (n = 5) group. The HD group display clearly elevated DynB in both high medial and lateral SN (arrows). (C) The increase in DynB peak intensity was most pronounced in the lateral tier of SN. Values are expressed as mean % peak area of intact SN ± SEM (p<0.01 HD vs. LD, HD vs. LC). (D) Correlation of Dyn B peak intensities and the cumulative dyskinesia score. Scale bar = 2 mm.

The peak corresponding to Dyn B displayed 44% higher peak area intensities in the SN ipsilateral to the DA-denervating lesion of HD animals (Fig. 4A). No side differences in Dyn B peak intensities were detected in the SN of lesion-only controls or low-dyskinetic animals. The MALDI ion images revealed that Dyn B distribution was mainly located to the medial aspect of SN in low-dyskinetic and lesion control, whereas L-DOPA-induced Dyn B peak intensity in high-dyskinetic animals was most pronounced in the lateral tier of SN reaching a 1.75-fold increase on the lesioned side (Fig. 4B and C, p<0.01 HD vs. LD and LC). Low levels of Dyn B were also detected in the interpeduncular nucleus and the part of hippocampus adjacent to the ventral midbrain.

Correlation analysis was carried out in order to assess whether the severity of dyskinesia was associated with nigral levels of Dyn B. In the whole SN, Dyn B intensities displayed a weak but significant correlation to the cumulative dyskinesia score (p <0.05, R = 0.72). Regional analysis revealed that Dyn B peak intensities in the lateral SN were positively correlated to the cumulative dyskinesia score (Fig. 4D, p<0.01, R = 0.80), whereas Dyn B peak intensity in the medial SN part alone showed no correlation (p = 0.30, R = 0.36). The Dyn B peak intensities in the lateral SN correlated equally well with all dyskinesia subtypes individually (data not shown), but best to the cumulative sum of all dyskinesia scores. Animals that displayed predominantly axial, limb, and orolingual dyskinesias had a similar Dyn B distribution in SN as rats that mainly displayed rotational/locomotive and axial dyskinesia.

### Alpha-neoendorphin peak intensity correlates with dyskinesia severity

**Figure 5:**
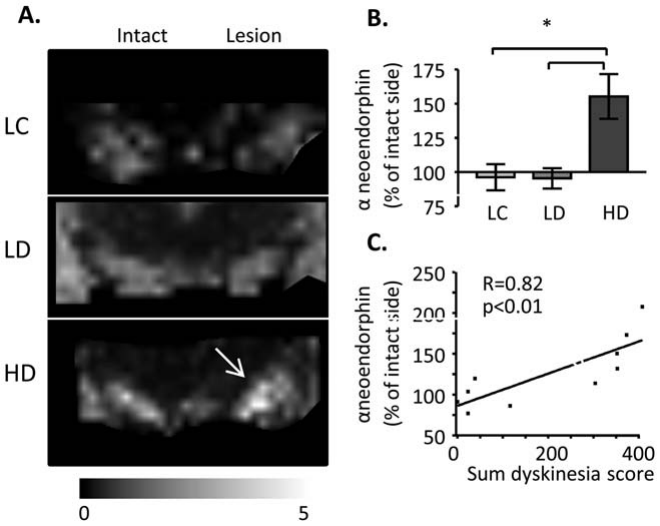
MALDI ion images of alpha-neoendorphin. (A) Representative MALDI IMS ion images of aNeo. (B) The increase in aNeo peak intensities were most pronounced in the lateral tier of SN (arrow). Values are expressed as mean % peak area of intact SN ± SEM (*p<0.05 for HD vs. LD, HD vs. LC). (C) Simple regression analysis of aNeo peak intensities and the cumulative dyskinesia score. Scale bar = 2 mm.

MALDI IMS ion images revealed low aNeo peak intensities in the SN, interpeduncular nucleus and hippocampus of lesion control and low-dyskinetic animals (Fig. 5A). Similarly to Dyn B, aNeo peak intensities were elevated 26% as measured in the whole medial and lateral part of SN ipsilateral to the DA-denervating lesion of HD animals. Regional analysis showed that the increase of aNeo peak intensity (54%) was most pronounced in the lateral part of SN in high dyskinetic animals compared with the low dyskinetic and lesion control animals (Fig. 5B; p<0.01 HD vs. LD, and HD vs. LC). Furthermore, aNeo peak intensity in the lateral part of SN positively correlated with the cumulative dyskinesia score reaching a 2-fold difference compared with the intact side in animals with severe dyskinesia (Fig. 5C; p<0.01; R = 0.82). Similarly to DynB the aNeo intensity in the whole SN displayed a weaker but still significant correlation (p<0.05, R = 0.74) whereas the intensity in the medial part alone did not correlate to the cumulative dyskinesia score (p = 0.47, R = 0.24).

### Dynorphin metabolites and dyskinesia

**Figure 6:**
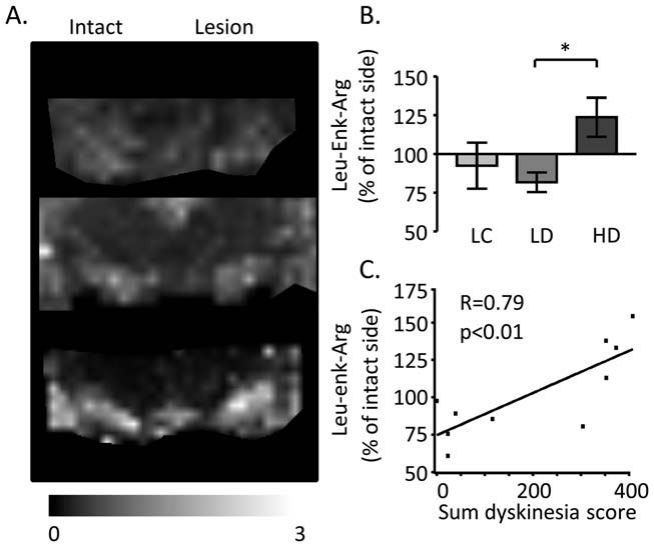
MALDI imaging of Leu-Enk-Arg. (A) Representative MALDI IMS ion images of and leu-enk-arg. (B) The increase in Leu-Enk-Arg peak intensities were most pronounced in the lateral tier of SN (arrow). Values are expressed as mean % peak area of intact SN ± SEM (*p<0.05 for HD vs. LD). (C) Simple regression analysis of Leu-Enk-Arg peak intensities and the cumulative dyskinesia score. Scale bar  = 2 mm.

**Figure 7:**
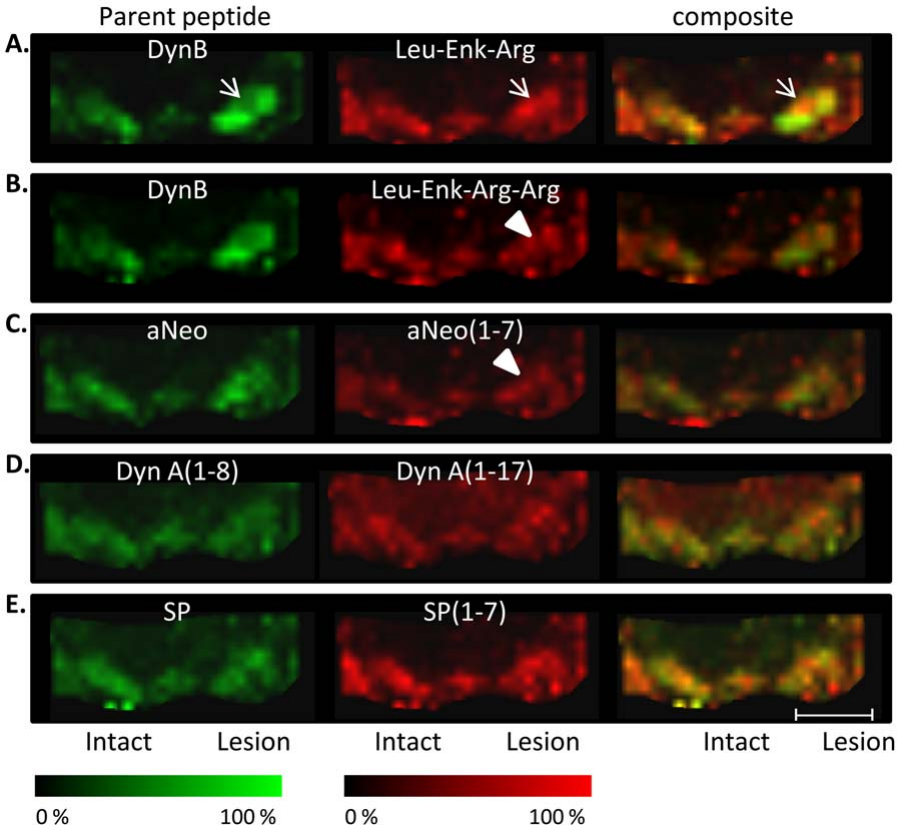
MALDI IMS ion images of peptides. (A) Composite MALDI IMS ion images from one single section of a high-dyskinetic animal reveal regional a distinct regional discrepancy between locally low Dyn B (green) and corresponding high Leu-Enk-Arg (red) levels in the dorsolateral SN (arrow). (B, C) Dynorphin metabolites Leu-Enk-Arg-Arg and aNeo(1–7) were also upregulated in the lateral, but not medial part of SN in HD animals (arrowhead). (D) Nigral levels of DynA (1–8), DynA (1–17), and Substance P (E) were not associated with any treatment-induced changes. Scale bar  = 2 mm.

In order to enable the study of dynorphin metabolites with MALDI IMS, the brains were removed 60 minutes after the last L-DOPA injection. Several peaks from dynorphin metabolic fragments displayed significantly higher peak intensities only in the lateral SN of HD rats, including Leu-Enk-Arg at m/z 712 (Fig. 6A and B; p<0.05 HD vs. LD). The Leu-Enk-Arg ion images revealed a similar distribution pattern as DynB and aNeo, with Leu-Enk-Arg mainly localized to SN, interpeduncular nucleus and the parts of hippocampus flanking the ventral midbrain. Furthermore, Leu-Enk-Arg peak intensities in the lateral but not medial part of SN were positively correlated with the cumulative dyskinesia score (Fig. 6C; p<0.01, R = 0.79). Multiplexing the ion images of both Dyn B and Leu-Enk-Arg revealed that the regional distribution of the peptides displayed an inverted pattern, as small regions with elevated Leu-Enk-Arg peak intensity showed corresponding lower Dyn B peak intensity in the same distinct area of the dorsolateral SN (Fig. 7A).

One putative dynorphin metabolic fragment corresponding to Leu-Enk-Arg-Arg (m/z 867) was upregulated by 54% in the HD group compared to the LD group but showed no significant correlation with LID severity (Fig. 7B; expressed as % of intact side, mean ± SEM; HD 170±18, LD 110±15; p<0.01 HD vs. LD; p = 0.07 and correlation coefficient R = 0.59;). Additionally, a putative metabolic product of aNeo (aNeo (1–7); m/z 840) displayed a 45% elevation in peak intensity without a significant correlation with dyskinesia (Fig. 7C; mean ± SEM, HD 148±12, LD 102±15; p<0.05; p = 0.08 and correlation coefficient R = 0.54). Notably, we could not detect any changes in peak intensities for Dyn A(1–17, m/z 2145), Dyn A(1–8, m/z 981), and substance P (m/z 1347; Fig. 7 D, E). Leu-Enk (m/z 555) was not detected in this study most probably due to the limit of detection in the low molecular weight region. Another way to effectively reduce opioid receptor activation of dynorphins is by removing the first N-terminal tyrosine [35]. Metabolic fragments corresponding to the des-tyrosine forms of Dyn A(1–8), aNeo, and Dyn B were observed in SN but displayed no treatment-induced changes (Supplemental Fig. S2).

### Dyskinesia is associated with high levels of dynorphin B-immunoreactivity in the lateral SN

**Figure 8:**
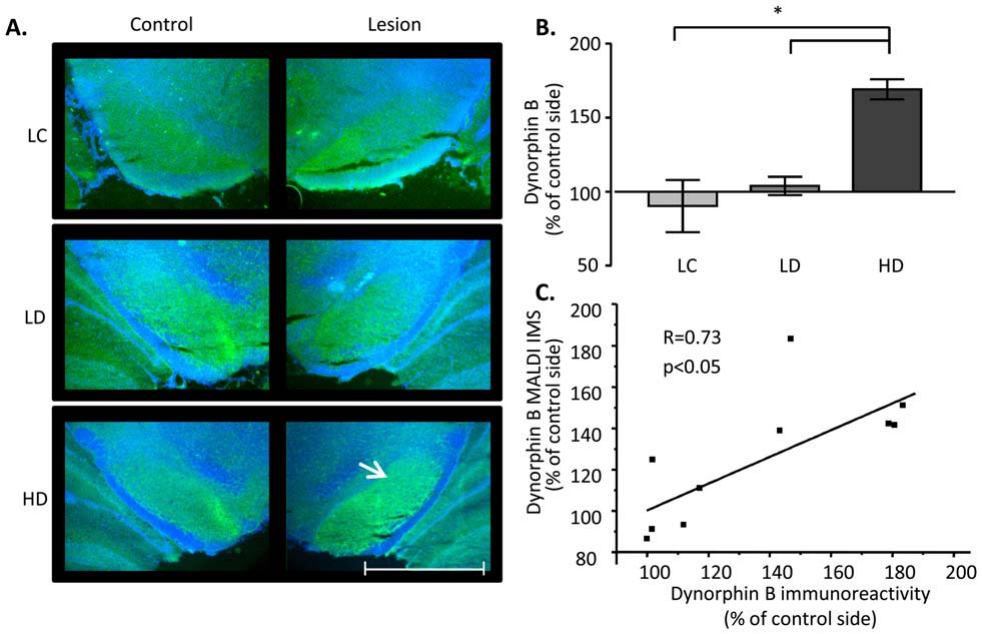
Immunohistochemistry of dynorphin B. (A) Immunohistochemical localization of DynB display higher immunolabeling of DynB-positive axons predominantly in the lateral SN of high dyskinetic animals (arrow). (B) Semi-quantization of DynB optical density measurements show elevated immunoreactivity in the lateral tier of SN. Values are expressed as mean % OD of intact SN ± SEM (p<0.001 HD vs. LD, HD vs. LC). (C) A linear correlation between the Dyn B peak intensity and the corresponding optical density of Dyn B immunoreactivity was observed. Scale bar  = 2 mm.

Confirming the MALDI IMS data, Dyn B-immunoreactive axons densely innervated the substantia nigra and the hippocampus adjacent to the ventral midbrain (cf. Fig. 8 and Fig. 4). In high-dyskinetic animals, Dyn B immunoreactivity was elevated by 63% in the lateral SN (Fig. 8B; p<0.001, HD vs. LD, and HD vs. LC group). A smaller increase by 39% was detected in the medial part of SN (p<0.05, HD vs. LD, mean % optical density ± SEM, HD 130±13, LD 93 ± 8). Indeed, Dyn B immunoreactivity in the medial part showed a weak but still significant correlation with cumulative dyskinesia score (p<0.05 R = 0.61, data not shown), however, the strongest correlation was localized to the lateral part of SN (p<0.001, R = 0.83, data not shown). This is strikingly similar to that observed from the MALDI IMS experiment (R = 0.80, p<0.01). Indeed, a linear correlation between the MALDI Dyn B peak intensity and the corresponding optical density of Dyn B immunoreactivity was observed (Fig. 8C; p<0.05, R = 0.73).

### Dynorphin peptide validation by IMS of Pdyn knockout mice

**Figure 9:**
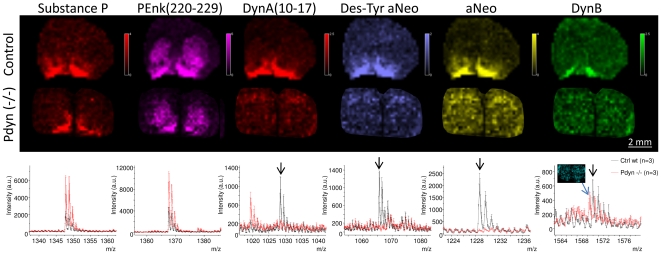
MALDI IMS of striatal sections reveal loss of dynorphins in Pdyn -/- knockout mice. (A) Substance P and dynorphin peptides are mainly localized to the nucleus accumbens and ventral pallidum of wild type (wt) normal control mice (top panel), whereas no dynorphin signal can be detected in the Pdyn-/- knockout mouse striatum (corresponding bottom panel). Enkephalin peptide PEnk(220–229) is also unaffected in the Pdyn-/- knockout mouse striatum. The intensity scalebars were set to the same absolute value in ion images from both knockout and control mice. (B) The loss of dynorphins, but not substance P and PEnk (220–229), in Pdyn-/- knockout mouse compared with wild type controls is evident by the average mass spectra from nucleus accumbens. Scale bar  = 2 mm.

MALDI imaging on striatal sections from wild type control and Pdyn knockout mice was performed for validation of dynorphin peptide identities (Fig. 9) [26]. Characteristic high levels were observed in wild type control mouse nucleus accumbens of several prodynorphin peptides, for example DynA (10–17), aNeo, DynB, and the metabolite desTyr-aNeo. Most peptide ion distribution patterns and levels were not different between wild type control striatum, for example substance P and PEnk 220–229. By contrast, no dynorphin peptide peaks could be detected in the striatum of Pdyn knockout mice (Fig. 9).

## Discussion

In the present study we demonstrate that L-DOPA-induced dyskinesia in a rat model of PD is associated with elevated levels of Dyn B and aNeo in the lateral, but not medial part of substantia nigra. Lower peak intensities of other dynorphins, such as Dyn A(1–8), Dyn A(1–17), and bNeo, were also detected by MALDI IMS but these were not associated with higher levels in dyskinetic animals. The predominant presence of aNeo and Dyn B in intact SN is in accordance with radioimmunoassay measurements from intact rat SN, where levels of alpha-neoendorphin and Dyn B were especially high followed by lower levels of Dyn A(1–8) and leu-enkephalin [16], [19], [20]. In addition, in the normal intact rat SN both aNeo and Dyn B peak intensities were particularly high in the ventromedial tier of SN similar to previously shown by immunohistochemistry [36]. Immunohistochemical localization of Dyn B was used as a second means of validation of the IMS data. Dyn B-immunoreactive axons densely innervated the substantia nigra with a spatial distribution that corresponded well to the distribution of the peptide peak as obtained by IMS, including strong Dyn B immunoreactivity observed in the lateral SN of high dyskinetic animals. Measurements of optical density of immunoreactivity in tissue does not reflect an absolutely quantitative measurement of neuropeptide levels, just as MALDI MS without the proper standard dilution curve can only approximate quantitation. Antibody specificity is a major issue particularly for neuropeptide analysis, however, by comparing immunoreactivity or peak intensity between the lesioned and the corresponding intact control side it is possible to obtain a quite reliable estimation of relative changes in peptide abundance. In this study the particular DynB antibody has been extensively validated and displays no cross-reactivity to other dynorphin peptides (19). Taken together with the observation of the close correlation between the dynorphin B peak intensity from MALDI IMS and dynorphin B immunoreactivity in the same animals (Fig. 8C; p<0.05, R = 0.73) present convincing evidence that the dynorphin B neuropeptide levels are increased in animals with severe dyskinesia.

**Figure 10:**
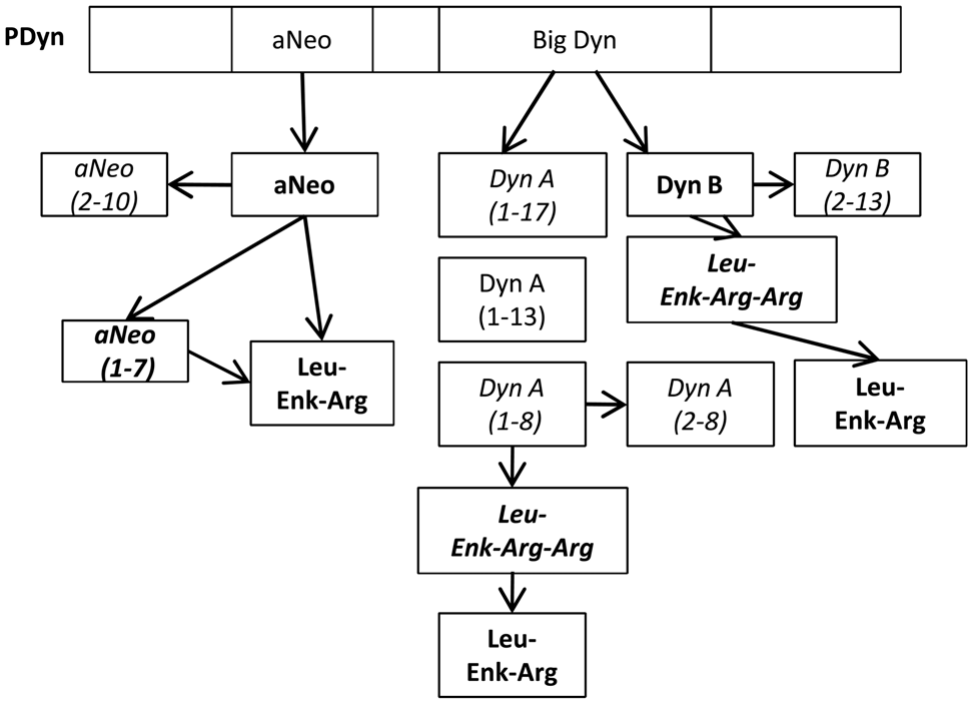
Schematic overview of peptides derived from Prodynorphin observed in SN. Peptides in bold were upregulated in high-dyskinetic animals. Peptides in italic were detected in this study but showed no treatment-related changes. The Leu-Enk-Arg peptide could be derived from the first 6 amino acids of several dynorphins, including aNeo, Dyn A(1–17), Dyn A(1–8) and Dyn B.

In order to measure not only the levels of neuroactive peptide by MALDI IMS but also dynorphin metabolites, the animals were sacrificed 60 minutes after the last L-DOPA injection at the time when the rats exhibit most dyskinesia (Fig. 2B). In contrast to most neurotransmitters, the opioid peptides are not recycled and are inactivated by proteases or converted to other bioactive peptide products [37]. The main metabolite detected in this study was Leu-Enk-Arg that displayed high peak intensities in the lateral SN and a strong correlation with LID. MALDI IMS offers the ability to reveal regional overlaps of protein localization by means of visualization of several peaks and their spatial distribution concurrently. Visualizing both Leu-Enk-Arg and Dyn B revealed a striking site-specific expression with high Leu-Enk-Arg in areas where Dyn B peak intensities were low (Fig. 7A). A tantalizing explanation suggests that dynorphin B has been released in these areas, followed by metabolic conversion to Leu-Enk-Arg by for example the dynorphin-converting enzyme [38]. In addition, the regional localization in nigra corresponds to striatonigral efferents from the lateral, but not medial striatum [16]. The possible conversion of Dyn B to Leu-Enk-Arg is highly interesting since the cleavage of Dyn B into Leu-Enk-Arg enhance the receptor binding specificity to delta opioid receptors [39]. Indeed, Leu-Enk-Arg is one of the main metabolites of synthetic Dyn B as detected in substantia nigra enzyme extracts [40]. The Leu-Enk-Arg peptide could be derived from the first 6 amino acids of several dynorphins, including aNeo, Dyn A(1–17), Dyn A(1–8) and Dyn B. The seventh amino acid differentiate between aNeo(1–7; YGGFLRK) and Leu-Enk-Arg-Arg (YGGFLRR) derived from either Dyn A(1–7) and/or Dyn B(1–7), both of which displayed elevations in high-dyskinetic animals. Taken together, this suggests that both Dyn B and aNeo are metabolized through cleavage into Leu-Enk-Arg (Fig. 10). Proteolytic degradation of samples is always a major concern in proteomic studies and indeed it has been shown that prolonged dissection times promote the degradation of dynorphins [41]. Several nigral peptide ion peaks correspond to the removal of the initial tyrosine that is important for opioid receptor binding and activation (Supplemental Fig. S2). Contrary to what was observed in the striatum, none of the des-tyrosine dynorphin peptides were affected by L-DOPA or associated with dyskinesia[42]. In the present study no other peaks corresponding to a step-wise amino acid removal from the either N- or C-terminal end of Dyn B or aNeo, suggesting that general proteolysis was minimal.

Clinical studies have suggested that an early initiation of L-DOPA- treatment in PD would relieve PD symptoms beyond the immediate effect of L-DOPA repletion and that this long-duration motor improvement is sustained for hours to days after cessation of L-DOPA treatment [43], [44], [45]. Different mechanisms have been suggested from L-DOPA-induced elevations of GDNF and BDNF to a neuroprotective role of L-DOPA itself, however so far we have not had an animal model in which to study such compensatory mechanisms. In this study, a worsening of “PD symptoms” was detected as an increase in left forelimb asymmetry in untreated lesion control animals over time. By contrast, this worsening was not seen in L-DOPA-treated animals and in low-dyskinetic animals this worsening was not only prevented but slightly reversed (Fig. 2D). We propose that differentiating between low- and high-dyskinetic animals will allow us to study the molecular mechanisms underlying the long-duration motor improvement in the rat model of LID in PD. L-DOPA treatment was initiated 3 weeks after DA-denervation, which is relatively early and thus the effect on parkinsonian forelimb use might reflect an early compensatory mechanism in form of protection of DA-cell degeneration possibly through a non-opiate receptor-mediated effect of dynorphins on nigral cells [46], [47].

L-DOPA-induced dyskinesia in patients with PD has been linked to elevated preproenkephalin (PPE-A) and PDyn mRNA levels in the striatum [48], [49], [50]. Especially high striatal PDyn mRNA levels have been strongly associated with LID in animal models of dyskinesias and PD, including primates, monkeys and rodents[51]. Traditionally the increase in PDyn mRNA is proposed to reflect an increased activity of striatonigral neurons of the direct basal ganglia pathway in dyskinesia [52]. In line with this, antisense-mediated fosB knockdown was paralleled with a reduction in PDyn mRNA levels and blocked LID development over time [53]. However, until now the identity of what prodynorphin peptide products were unknown and insight into opioid transmission in LID was mainly based on receptor-binding studies. The endogenous dynorphins mainly display high affinity to the kappa opioid receptor (KOP), but can also interact with the other classical opioid receptors mu (MOP) and delta (DOP) with slightly lower binding affinities [54], [55]. Receptor binding studies have suggested activation of both KOP and MOP receptors in the substantia nigra of rat and monkey models of LID in PD [56], [57]. In both studies a significant negative correlation between dyskinesia and KOP and MOP receptor-binding in basal ganglia output structures indicated increased striatonigral opioid transmission in dyskinesia. This is in line with the results of the present study, since although all dynorphins display high KOP receptor binding, both Dyn B and aNeo can interact with the other opiate receptors and in particular aNeo displays the least selectivity being active in the nanomolar range at both DOPr and MOPr [39], [58].

Clinical and preclinical studies of the effects of opioid agonists and antagonists on LID have shown contradictory results[51]. Systemic administration of non-selective opiate antagonists has been associated with both increases and decreases of LID in parkinsonian monkeys and patients [8], [9], [10], [12], [59]. Specific kappa-agonist U50-488 has shown promising anti-dyskinetic effects in both rat and monkey models of PD, however in monkey the anti-parkinsonian symptomatic relief by L-DOPA was abolished and furthermore, severe side effects in form of sedation and vomiting reduced the therapeutically value [6], [7]. The present study indicates that prodynorphin processing in the striatonigral projection neurons leads to peptides that are not the most effective kappa agonists and may result in abnormal signaling through the delta and mu opioid receptor instead. Indeed, both DOPr and MOPr-antagonists attenuate LID in the MPTP-lesioned marmoset [5].

For the first time it has been possible to demonstrate that chronic intermittent L-DOPA at a dose regime that is known to cause an upregulation of striatal PDyn mRNA levels is also associated with a concomitant elevation in dynorphin B and alpha-neoendorphin peptide levels in one of the basal ganglia output structures, the substantia nigra. Furthermore, using imaging mass spectrometry it was possible to localize the predominant area affected to the lateral part of SN, where the two opioid peptide peak intensities were most strongly associated with the severity of L-DOPA-induced dyskinesia. By contrast, the Dyn A-related peptides, which have the highest affinity to KOP receptors, were detected at low levels but did not show any treatment- or LID-associated changes. The main dynorphin metabolite detected, Leu-Enk-Arg that has even less affinity to KOP receptors, also displayed a strong correlation between the peak intensities in the lateral SN and LID severity. So far no subtype-selective opioid antagonists have been evaluated in PD patients with LID; however, our data suggest that both DOP and MOP receptor specific antagonist could be of potential interest.

## Supporting Information

Figure S1 **MS/MS analyses of dynorphins.** (A) Examples of fragmentation spectra of endogenous dynorphin B and (B) alpha-neoendorphin. Similar results were obtained for Leu-Enk, Leu-Enk-Arg, dynorphin A(10–17), and alpha-neoendorphin (2–8). For peptide identification the database search was made with no enzyme/unspecific cleavage, and no fixed or variable modifications.
(TIF)

Figure S2 **MALDI IMS of putative des-tyrosine dynorphins.** (A) Composite MALDI IMS ion images from one single section of a high dyskinetic animal reveal an overlap in regional distribution of DynA(1–8), visualized in green, and its potential des-tyrosine fragment DynA(2–8) in red.(B) A similar pattern was observed for aNeo(green) and its des-tyrosine fragment aNeo(2–10) (red), and(C) DynB (green) and its corresponding des-tyrosine peptide DynB(2–13) (red), however no treatment-induced changes were detected for the des-tyrosine peptides. Des-tyrosine aNeo (2–10) has been identified by LC-MS/MS. Scale bar = 2 mm.
(TIF)

## References

[1] Schapira AH, Emre M, Jenner P, Poewe W (2009). Levodopa in the treatment of Parkinson's disease.. Eur J Neurol.

[2] Stocchi F, Jenner P, Obeso JA (2010). When do levodopa motor fluctuations first appear in Parkinson's disease?. Eur Neurol.

[3] Piccini P, Weeks RA, Brooks DJ (1997). Alterations in opioid receptor binding in Parkinson's disease patients with levodopa-induced dyskinesias.. Ann Neurol.

[4] Fox SH, Johnston TH, Brotchie JM, Dean RL, Bilsky EJ, Negus SS (2009). Effects of Opioid Antagonists on L-DOPA-Induced Dyskinesia in Parkinson's Disease.. Opiate Receptors and Antagonists.

[5] Henry B, Fox SH, Crossman AR, Brotchie JM (2001). Mu- and delta-opioid receptor antagonists reduce levodopa-induced dyskinesia in the MPTP-lesioned primate model of Parkinson's disease.. Exp Neurol.

[6] Marin C, Bove J, Bonastre M, Tolosa E (2003). Effect of acute and chronic administration of U50,488, a kappa opioid receptor agonist, in 6-OHDA-lesioned rats chronically treated with levodopa.. Exp Neurol.

[7] Cox H, Togasaki DM, Chen L, Langston JW, Di Monte DA (2007). The selective kappa-opioid receptor agonist U50,488 reduces L-dopa-induced dyskinesias but worsens parkinsonism in MPTP-treated primates.. Exp Neurol.

[8] Rascol O, Fabre N, Blin O, Poulik J, Sabatini U (1994). Naltrexone, an opiate antagonist, fails to modify motor symptoms in patients with Parkinson's disease.. Mov Disord.

[9] Samadi P, Gregoire L, Bedard PJ (2004). The opioid agonist morphine decreases the dyskinetic response to dopaminergic agents in parkinsonian monkeys.. Neurobiol Dis.

[10] Samadi P, Gregoire L, Bedard PJ (2003). Opioid antagonists increase the dyskinetic response to dopaminergic agents in parkinsonian monkeys: interaction between dopamine and opioid systems.. Neuropharmacology.

[11] Klintenberg R, Andren PE (2005). Altered extracellular striatal in vivo biotransformation of the opioid neuropeptide dynorphin A(1-17) in the unilateral 6-OHDA rat model of Parkinson's disease.. J Mass Spectrom.

[12] Fox S, Silverdale M, Kellett M, Davies R, Steiger M (2004). Non-subtype-selective opioid receptor antagonism in treatment of levodopa-induced motor complications in Parkinson's disease.. Mov Disord.

[13] El Atifi-Borel M, Buggia-Prevot V, Platet N, Benabid AL, Berger F (2009). De novo and long-term l-Dopa induce both common and distinct striatal gene profiles in the hemiparkinsonian rat.. Neurobiol Dis.

[14] Cenci MA, Lee CS, Bjorklund A (1998). L-DOPA-induced dyskinesia in the rat is associated with striatal overexpression of prodynorphin- and glutamic acid decarboxylase mRNA.. Eur J Neurosci.

[15] Vincent S, Hokfelt T, Christensson I, Terenius L (1982). Immunohistochemical evidence for a dynorphin immunoreactive striato-nigral pathway.. Eur J Pharmacol.

[16] Christensson-Nylander I, Herrera-Marschitz M, Staines W, Hokfelt T, Terenius L (1986). Striato-nigral dynorphin and substance P pathways in the rat. I. Biochemical and immunohistochemical studies.. Exp Brain Res.

[17] Weber E, Roth KA, Barchas JD (1982). Immunohistochemical distribution of alpha-neo-endorphin/dynorphin neuronal systems in rat brain: evidence for colocalization.. Proc Natl Acad Sci U S A.

[18] Christensson-Nylander I, Terenius L (1985). Dynorphin peptides in human substantia nigra.. Neuropeptides.

[19] Trujillo KA, Day R, Akil H (1990). Regulation of striatonigral prodynorphin peptides by dopaminergic agents.. Brain Res.

[20] Zamir N, Skofitsch G, Bannon MJ, Helke CJ, Kopin IJ (1984). Primate model of Parkinson's disease: alterations in multiple opioid systems in the basal ganglia.. Brain Res.

[21] Caprioli RM, Farmer TB, Gile J (1997). Molecular imaging of biological samples: localization of peptides and proteins using MALDI-TOF MS.. Anal Chem.

[22] Andersson M, Groseclose MR, Deutch AY, Caprioli RM (2008). Imaging mass spectrometry of proteins and peptides: 3D volume reconstruction.. Nat Methods.

[23] Paxinos G, Watson C (2007). The Rat Brain in Stereotaxic Coordinates.

[24] Schallert T, Fleming SM, Leasure JL, Tillerson JL, Bland ST (2000). CNS plasticity and assessment of forelimb sensorimotor outcome in unilateral rat models of stroke, cortical ablation, parkinsonism and spinal cord injury.. Neuropharmacology.

[25] Tillerson JL, Cohen AD, Philhower J, Miller GW, Zigmond MJ (2001). Forced limb-use effects on the behavioral and neurochemical effects of 6-hydroxydopamine.. J Neurosci.

[26] Sharifi N, Diehl N, Yaswen L, Brennan MB, Hochgeschwender U (2001). Generation of dynorphin knockout mice.. Brain Res Mol Brain Res.

[27] Norris JL, Cornett DS, Mobley JA, Andersson M, Seeley EH (2007). Processing MALDI Mass Spectra to Improve Mass Spectral Direct Tissue Analysis.. Int J Mass Spectrom.

[28] Li H, Chen S, Hong D, Li M, Shyr Y, Mass Spectrometry Binning Software GAB http://www.vicc.org/biostatistics/download/MassSpec/pBinWin.rar.

[29] Bergstrom L, Christensson I, Folkesson R, Stenstrom B, Terenius L (1983). An ion exchange chromatography and radioimmunoassay procedure for measuring opioid peptides and substance P.. Life Sci.

[30] Benjamini Y, Hochberg Y (1995). Controlling the false discovery rate: a practical and powerful approach to multiple testing..

[31] Team RDC (2008). R: A language and environment for statistival computing..

[32] Smyth GK (2004). Linear models and empirical bayes methods for assessing differential expression in microarray experiments.. Stat Appl Genet Mol Biol.

[33] Che FY, Zhang X, Berezniuk I, Callaway M, Lim J (2007). Optimization of neuropeptide extraction from the mouse hypothalamus.. J Proteome Res.

[34] Fricker LD (2010). Analysis of mouse brain peptides using mass spectrometry-based peptidomics: implications for novel functions ranging from non-classical neuropeptides to microproteins.. Mol Biosyst.

[35] Walker JM, Tucker DE, Coy DH, Walker BB, Akil H (1982). Des-tyrosine-dynorphin antagonizes morphine analgesia.. Eur J Pharmacol.

[36] Weber E, Barchas JD (1983). Immunohistochemical distribution of dynorphin B in rat brain: relation to dynorphin A and alpha-neo-endorphin systems.. Proc Natl Acad Sci U S A.

[37] Nyberg F, Hallberg M (2007). Peptide conversion--a potential pathway modulating G-protein signaling.. Curr Drug Targets.

[38] Silberring J, Nyberg F (1989). A novel bovine spinal cord endoprotease with high specificity for dynorphin B.. J Biol Chem.

[39] Mansour A, Hoversten MT, Taylor LP, Watson SJ, Akil H (1995). The cloned mu, delta and kappa receptors and their endogenous ligands: evidence for two opioid peptide recognition cores.. Brain Res.

[40] Sandin J, Tan-No K, Kasakov L, Nylander I, Winter A (1997). Differential metabolism of dynorphins in substantia nigra, striatum, and hippocampus.. Peptides.

[41] Nylander I, Stenfors C, Tan-No K, Mathe AA, Terenius L (1997). A comparison between microwave irradiation and decapitation: basal levels of dynorphin and enkephalin and the effect of chronic morphine treatment on dynorphin peptides.. Neuropeptides.

[42] Hanrieder J, Karlsson A, Falth M, Eriksson-Mammo S, Bergquist J (2011). L-DOPA-induced dyskinesia is associated with regional increase of striatal dynorphin peptides as elucidated by imaging mass spectrometry.. Mol Cell Proteomics *in press*.

[43] Nutt JG, Carter JH, Woodward WR (1995). Long-duration response to levodopa.. Neurology.

[44] Quattrone A, Zappia M, Aguglia U, Branca D, Colao R (1995). The subacute levodopa test for evaluating long-duration response in Parkinson's disease.. Ann Neurol.

[45] Chan PL, Nutt JG, Holford NH (2007). Levodopa slows progression of Parkinson's disease: external validation by clinical trial simulation.. Pharm Res.

[46] Brotchie J, Fitzer-Attas C (2009). Mechanisms compensating for dopamine loss in early Parkinson disease.. Neurology.

[47] Liu B, Qin L, Yang SN, Wilson BC, Liu Y (2001). Femtomolar concentrations of dynorphins protect rat mesencephalic dopaminergic neurons against inflammatory damage.. J Pharmacol Exp Ther.

[48] Nisbet AP, Foster OJ, Kingsbury A, Eve DJ, Daniel SE (1995). Preproenkephalin and preprotachykinin messenger RNA expression in normal human basal ganglia and in Parkinson's disease.. Neuroscience.

[49] Calon F, Birdi S, Rajput AH, Hornykiewicz O, Bedard PJ (2002). Increase of preproenkephalin mRNA levels in the putamen of Parkinson disease patients with levodopa-induced dyskinesias.. J Neuropathol Exp Neurol.

[50] Henry B, Duty S, Fox SH, Crossman AR, Brotchie JM (2003). Increased striatal pre-proenkephalin B expression is associated with dyskinesia in Parkinson's disease.. Exp Neurol.

[51] Fox SH, Johnston TH, Brotchie JM, Dean RL, Bilsky EJ, Negus SS (2009). Effects of Opioid Antagonists on L-DOPA-Induced Dyskinesia in Parkinson's Disease..

[52] Obeso JA, Rodriguez-Oroz MC, Rodriguez M, DeLong MR, Olanow CW (2000). Pathophysiology of levodopa-induced dyskinesias in Parkinson's disease: problems with the current model.. Ann Neurol.

[53] Andersson M, Hilbertson A, Cenci MA (1999). Striatal fosB expression is causally linked with l-DOPA-induced abnormal involuntary movements and the associated upregulation of striatal prodynorphin mRNA in a rat model of Parkinson's disease.. Neurobiol Dis.

[54] Merg F, Filliol D, Usynin I, Bazov I, Bark N (2006). Big dynorphin as a putative endogenous ligand for the kappa-opioid receptor.. J Neurochem.

[55] Schwarzer C (2009). 30 years of dynorphins--new insights on their functions in neuropsychiatric diseases.. Pharmacol Ther.

[56] Johansson PA, Andersson M, Andersson KE, Cenci MA (2001). Alterations in cortical and basal ganglia levels of opioid receptor binding in a rat model of l-DOPA-induced dyskinesia.. Neurobiol Dis.

[57] Aubert I, Guigoni C, Li Q, Dovero S, Bioulac BH (2007). Enhanced preproenkephalin-B-derived opioid transmission in striatum and subthalamic nucleus converges upon globus pallidus internalis in L-3,4-dihydroxyphenylalanine-induced dyskinesia.. Biol Psychiatry.

[58] Schulz R, Wuster M, Herz A (1982). Endogenous ligands for kappa-opiate receptors.. Peptides.

[59] Klintenberg R, Svenningsson P, Gunne L, Andren PE (2002). Naloxone reduces levodopa-induced dyskinesias and apomorphine-induced rotations in primate models of parkinsonism.. J Neural Transm.

