# Supplementary figures and images for: Imaging Mass Spectrometry Reveals Elevated Nigral Levels of Dynorphin Neuropeptides in L-DOPA-Induced Dyskinesia in Rat Model of Parkinson's Disease
Source: PLoS One. 2011 Sep 30;6(9):e25653. doi: 10.1371/journal.pone.0025653 (PMC3184165; doi:10.1371/journal.pone.0025653)

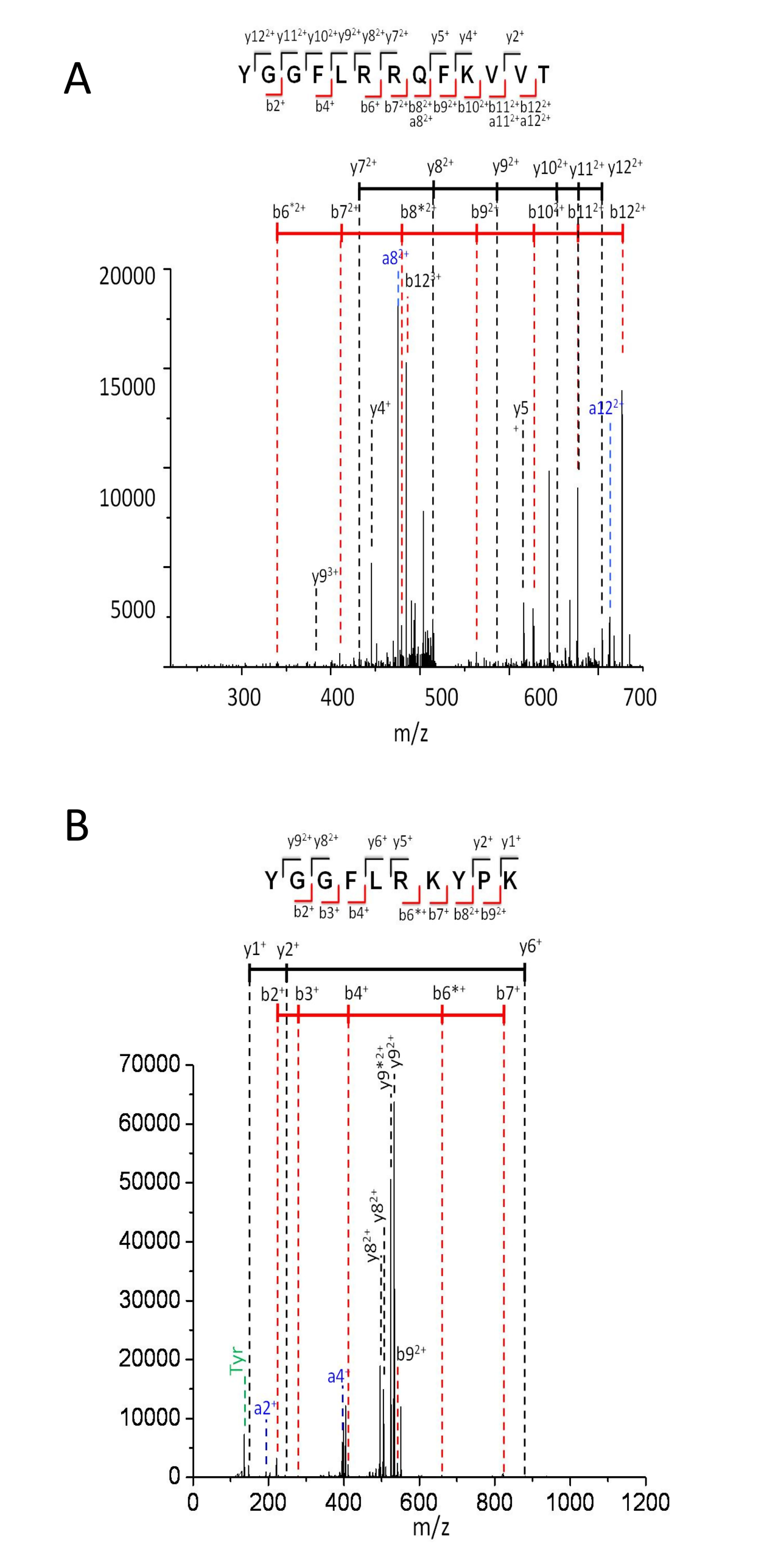

Supplement: Figure S1 — MS/MS analyses of dynorphins. (A) Examples of fragmentation spectra of endogenous dynorphin B and (B) alpha-neoendorphin. Similar results were obtained for Leu-Enk, Leu-Enk-Arg, dynorphin A(10–17), and alpha-neoendorphin (2–8). For peptide identification the database search was made with no enzyme/unspecific cleavage, and no fixed or variable modifications. (TIF) [file pone.0025653.s001.tif]

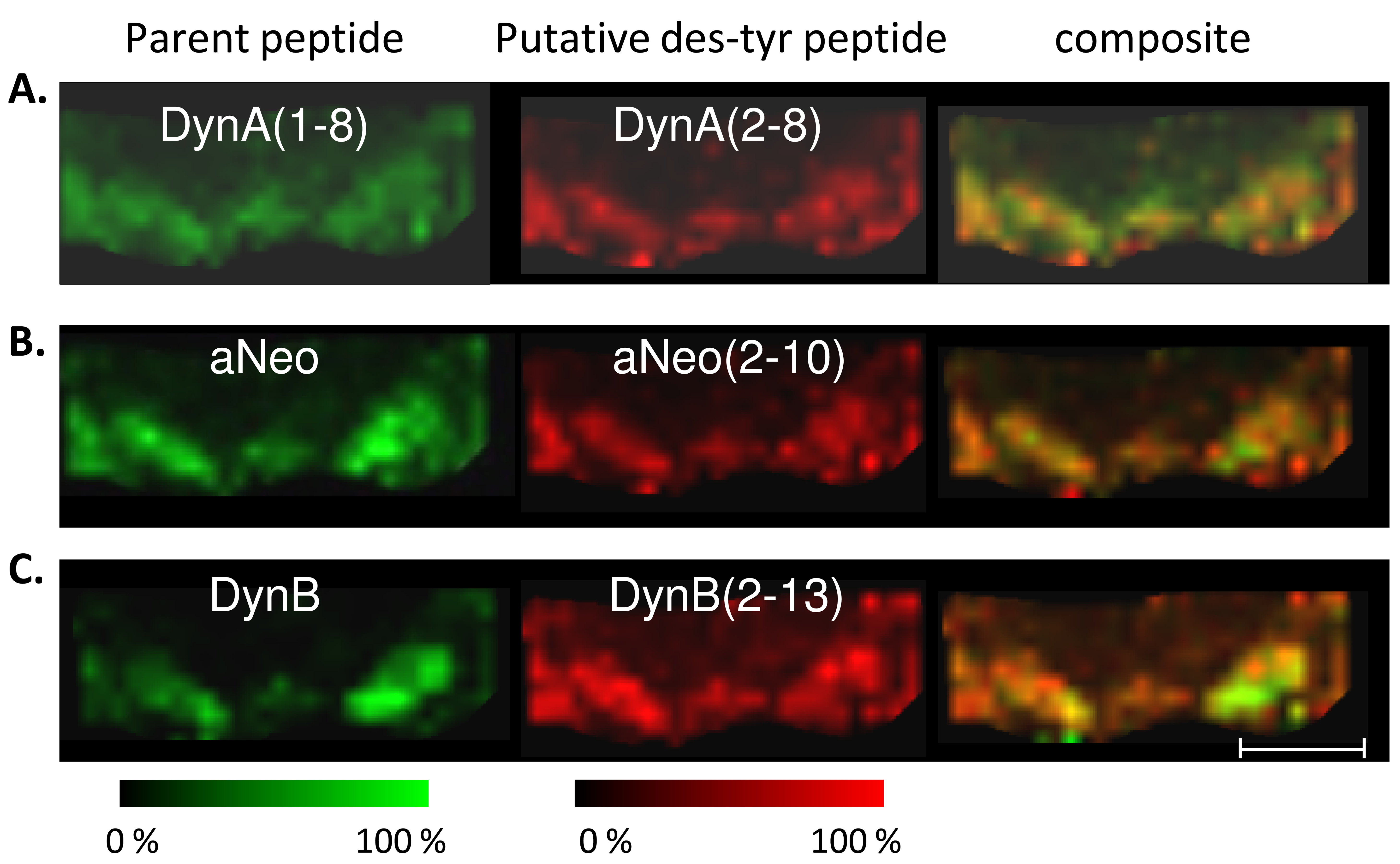

Supplement: Figure S2 — MALDI IMS of putative des-tyrosine dynorphins. (A) Composite MALDI IMS ion images from one single section of a high dyskinetic animal reveal an overlap in regional distribution of DynA(1–8), visualized in green, and its potential des-tyrosine fragment DynA(2–8) in red.(B) A similar pattern was observed for aNeo(green) and its des-tyrosine fragment aNeo(2–10) (red), and(C) DynB (green) and its corresponding des-tyrosine peptide DynB(2–13) (red), however no treatment-induced changes were detected for the des-tyrosine peptides. Des-tyrosine aNeo (2–10) has been identified by LC-MS/MS. Scale bar = 2 mm. (TIF) [file pone.0025653.s002.tif]
